# Automatic interpretation of otoliths using deep learning

**DOI:** 10.1371/journal.pone.0204713

**Published:** 2018-12-17

**Authors:** Endre Moen, Nils Olav Handegard, Vaneeda Allken, Ole Thomas Albert, Alf Harbitz, Ketil Malde

**Affiliations:** 1 Institute of Marine Research, Bergen, Norway; 2 Department of Informatics, University of Bergen, Norway; Department of Agriculture and Water Resources, AUSTRALIA

## Abstract

The age structure of a fish population has important implications for recruitment processes and population fluctuations, and is a key input to fisheries-assessment models. The current method of determining age structure relies on manually reading age from otoliths, and the process is labor intensive and dependent on specialist expertise. Recent advances in machine learning have provided methods that have been remarkably successful in a variety of settings, with potential to automate analysis that previously required manual curation. Machine learning models have previously been successfully applied to object recognition and similar image analysis tasks. Here we investigate whether deep learning models can also be used for estimating the age of otoliths from images. We adapt a pre-trained convolutional neural network designed for object recognition, to estimate the age of fish from otolith images. The model is trained and validated on a large collection of images of Greenland halibut otoliths. We show that the model works well, and that its precision is comparable to documented precision obtained by human experts. Automating this analysis may help to improve consistency, lower cost, and increase the extent of age estimation. Given that adequate data are available, this method could also be used to estimate age of other species using images of otoliths or fish scales.

## Introduction

Age of fish is a key parameter in age-structured fisheries-assessment models. Age is usually considered as a discrete parameter (age group) that identifies the individual year class i.e. those originating from the spawning activity in a given year [1]. An individual is categorized as age group 0 from the first early larval stage, and all age groups increase their age at 1 January. Assessment models typically express the dynamics of the individual year class from the age when they recruit, through sexual maturation, reproduction, and throughout their life cycle [2]. The models are fitted to data originating from commercial catches and fisheries-independent surveys. A sampling program for a specific fish stock typically involves sampling throughout the year using several different types of fishing gears.

Fish age is typically estimated using samples of individual fish. Since fish growth and age-at-length varies in time and space (e.g., [3]), linked environmental variables such as temperature, food availability and morphology (e.g., fish length) cannot be reliably used as a proxy for age. Instead, age is determined from a subset of individuals and usually used in conjunction with length data, and information relating to the time and location of sampling [3]. The age is “read” from the annual zones in otoliths or fish scales. Although simple in principle, age reading depends on the correct identification of zonation patterns that may consist of both true annual zones, and zones representing other (unknown) temporal variation [1, 4]. The process is time consuming, requires a trained eye, and is uncertain. This uncertainty can be divided into accuracy and precision. Whereas reader precision and between-reader bias can be assessed from age-readings, the bias of the age estimator is difficult to estimate but may be assessed using a number of methods (e.g. radiochemical analyses, analysis of chemical tags, or tag-recapture experiments [5]).

Methods to automatically read otoliths have been proposed, but to date none have proven satisfactory. Fablet and Le Josse [6] investigated feature extraction from images of otoliths using statistical learning techniques, including both neural networks and support vector machines. They considered both biological features, including fish length, sex and catch date, and geometrical features, including shape and the opaque and translucent zonation patterns. Using both sets of features, they found that the models did not significantly improve predictions when compared to using just biological data. Robertson and Alexander [7] found that precision of predicting age of otoliths using neural networks from geometric features could be improved by using biological features, but the results obtained from neural networks were less precise than those obtained from experienced readers.

### Convolutional neural networks

Artificial neural networks are computational structures inspired by biological neural networks [8]. They consist of simple computational units referred to as *neurons*, organized in layers. The neuron parameters (or weights) are estimated by training the model using supervised learning. This process consists of two steps: i.) forward propagation, where the network makes a prediction based on the input, and ii) back propagation, where the network learns from its mistake by calculating the gradient of a loss function, and then uses the gradient to update the neuron weights.

In recent years, neural networks have become widely successful, especially in the field of image analysis. In 2016, the neural network designed by Krizhevsky et al. [9] was used to substantially improve the performance of an important benchmark task, object recognition, and the results were subsequently improved on by more refined network architectures [10–12], even to the point of rivaling human abilities. One important improvement was an increase in the number of layers; this is often referred to as *deep learning*.

The most remarkable feature of deep learning neural networks is perhaps their generality. With sufficient training data, they can be used to classify raw data (e.g. an array of pixels) directly i.e. no explicit design of low-level features is necessary. The network’s lower layers learn to distinguish between primitive features automatically, typically identifying sharp edges or color transitions. Subsequent layers then learn to recognize more abstract features as combinations of lower layer features, and finally merge this information to provide a high level classification. In a convolutional neural network (CNN), the layers are organized as a stack of convolutions, applying the same filters across the whole image. An important advantage of this approach is that the number of parameters to be learned is reduced, which again reduces the amount of data and computation necessary for training.

Here we explore whether a CNN can be used to reliably estimate the age of an otolith from an image. We implement a network architecture and train it on otolith images from Greenland halibut (*Reinhardtius hippoglossoides*). We then evaluate the precision of our classifier by comparing it to existing age estimates from human experts.

## Methods and materials

### Data collection and preprocessing

The data set consists of pre-existing images of otoliths from the Institute of Marine Research (IMR, Bergen, Norway) image archive. Fish otoliths were collected and photographed as part of the IMR data collection program for Greenland halibut on cruises between 2006 and 2017. The otolith-derived age data constitutes an important input to the stock assessment program, and represents a valuable source of historical information. The data set is comprised of 4109 images of otolith pairs and 657 images of single otoliths, totaling 8875 otoliths. As the present study only investigated historical pre-existing data, and did not involve the collection of new animals, ethics approval was not necessary.

**Fig 1:**
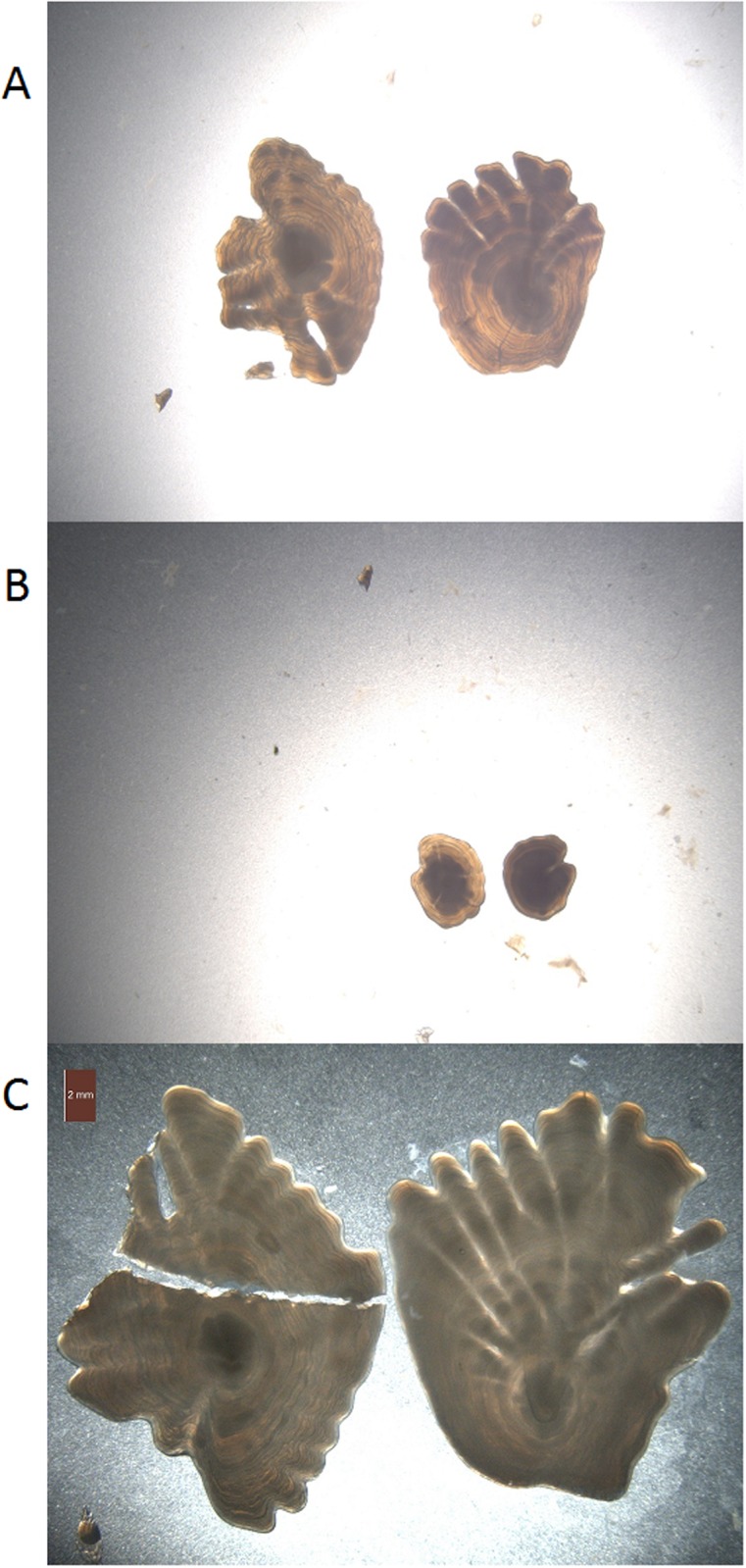
Example of otolith images. Otoliths can have loose fragments (A), vary in size (B), or be broken (C).

The process of reading the otoliths from the images is described in Albert et al. [13]. The images have a resolution of 2596 x 1944 pixels. During preparation and transportation, the otoliths were sometimes damaged or lost, which resulted in only one of the two otoliths being present. The images also varied in distance to object, lighting, and background. Examples of image variation and damaged otoliths are shown in Fig 1.

**Fig 2:**
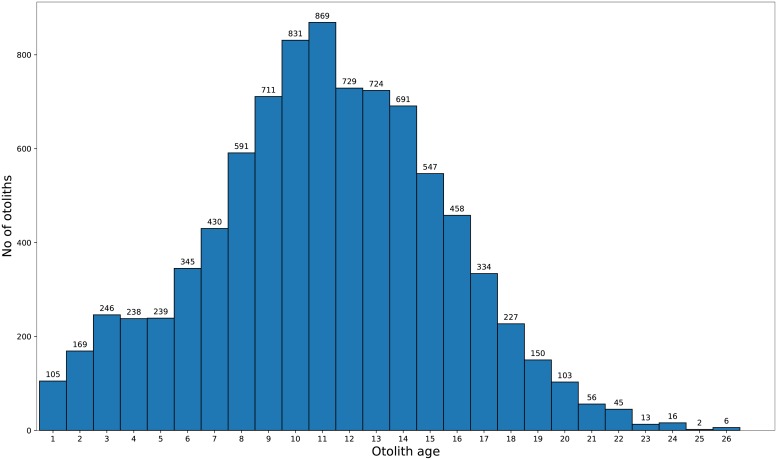
Age distribution of all 8875 images.

The age of each otolith pair had previously been estimated by one of two expert readers from the same lab (IMR facilities in Tromsø, Norway). The estimated age distribution for all 8875 images is shown in Fig 2. Until recently, there was no standardized method for the age reading of Greenland halibut otoliths. But as a result of two International Council for the Exploration of the Sea workshops [14, 15], two different methods were recommended, both of which resulted in reasonably accurate age estimates. The age estimates in this study were based on one of these methods, named the ‘whole right otolith’ method [13, 15, 16]. In a flatfish like Greenland halibut, the growth patterns differ between the two otoliths. While the left otoliths show a centric growth pattern, the right otoliths are clearly acentric. The longest growth axis from the center to the edge is therefore found in the right otoliths. This longest growth axis consistently shows more patterns attributable to annuli than any other growth axes of the whole left or right otoliths [13, 16]. Since reader ID is not recorded for each otolith, there is potential for reader-specific bias in the data.

**Fig 3:**
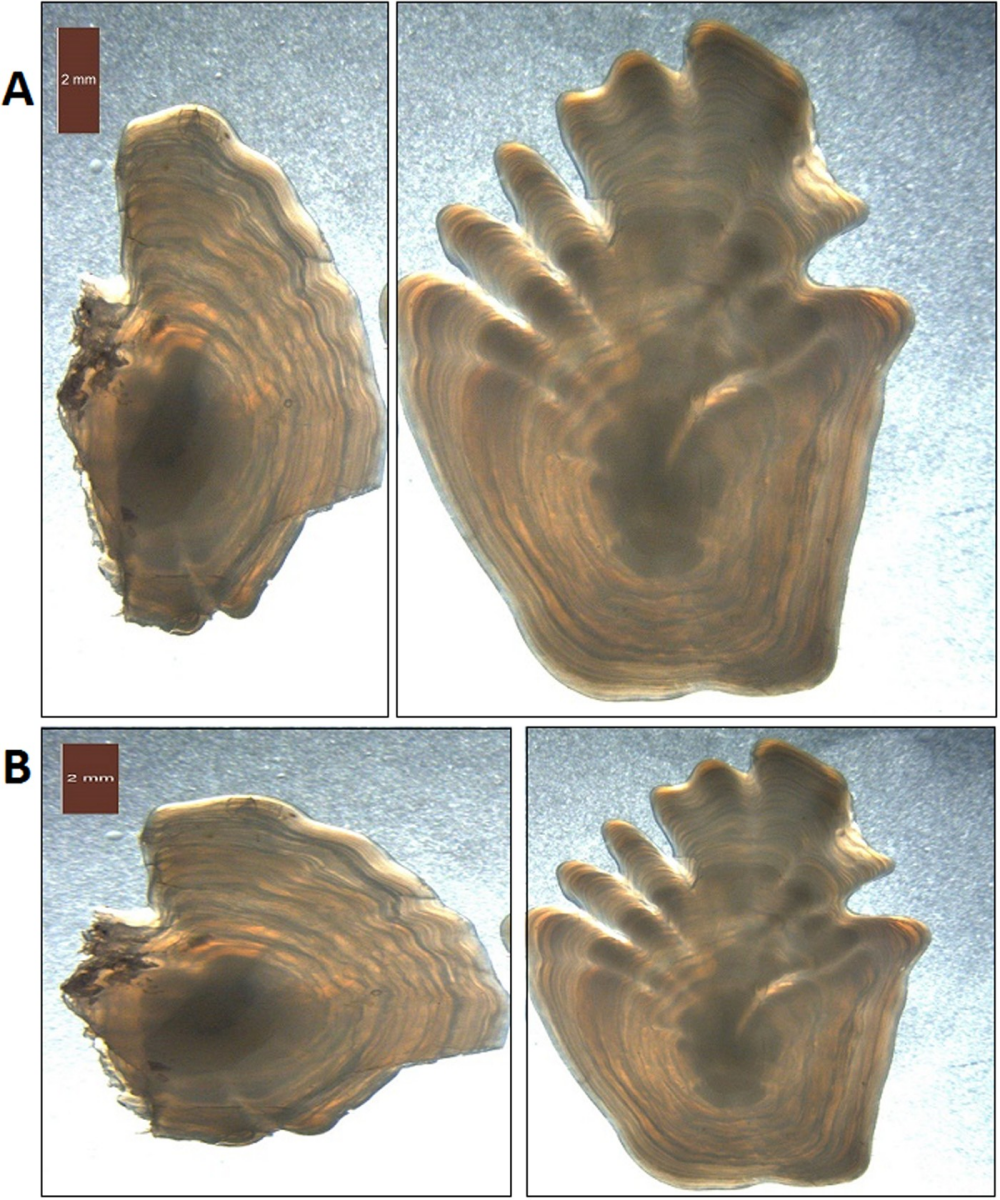
A pair of otoliths from 2014 with an estimated age of 13 years. Due to the size difference between the otoliths, the image was split with a substantial offset from the middle (A). There was also a small horizontal overlap causing a fragment of the right otolith to remain in the left image. Resizing causes stretching of the images (B), which is particularly evident in the image of the left otolith.

Prior to the analysis, the images of the paired otoliths were split, resulting in separate images of the left and right otoliths. Due to variation in the placement of the otoliths in the original images, the new split images were reviewed and the split adjusted manually. The horizontal position of the split varied by up to 350 pixels. In some cases, the otoliths overlapped horizontally, resulting in images containing a small fraction of the other otolith. This overlap was rarely more than 30 pixels. Finally, images of individual otoliths were rescaled to a standard size of 400 x 400 pixels. Although this caused images to be stretched or shrunk, CNNs have shown to be robust to random transformations [17, 18]. The process is illustrated in Fig 3. Information relating the paired otolith images to the separated right and left ototith images was retained in order to predict the age of the pairs, and evaluate the accuracy of predicting left and right otoliths.

### Convolutional neural network architecture

We used a classifier model based on the Inception v3 [19] model. This is a state of the art 48-layer architecture for image classification, and the successor to the network [20] that won the 2014 ImageNet competition [21]. There are several competing architectures, and variations of ResNet [10] (ResNet50, ResNet101, and ResNet152), Inception v4 [22], and DenseNet121 [23] were considered, but preliminary testing showed small differences in results, with the preliminary performance of most configurations varying less than 10%.

Inception v3 classifies images with a size of 299 x 299 pixels into one of 1000 categories. To use this model to analyze the otolith images, some modifications to the network were necessary. First, the input layer was scaled to match the image size of 400 x 400 pixels. Since age estimation is a regression problem, the output layer was changed from a 1000-dimension output vector, representing class probabilities, to a single numeric output. Finally, the objective (or loss) function, used in optimization, was changed from cross entropy to mean squared error (MSE) defined as

$$\begin{array}{c} MSE=\frac{1}{n}\sum_{t=1}^{n}{\left(\hat{y_{t}}-y_{t}\right)}^{2} \end{array}$$ (1)

where $\hat{y_{t}}$ is the CNN prediction and *y*_*t*_ is the read age, and *n* is the number of predictions.

The CNN layers were loaded with pre-trained (using ImageNet data) and publicly available weights, as opposed to using random initialization, which is inefficient [24]. All layers were set to trainable i.e. the values of the individual neuron weights were updated during training.

### Training the convolutional neural network

The CNN was implemented using the standard software packages Keras [25] with TensorFlow [26], and computation was performed using CUDA version 9.1 and CuDNN with nVidia (nVidia Corp., Santa Clara, California) P100 accelerator cards.

The data set was split into training, validation and testing sets, containing 92%, 4% and 4% of the images, respectively. The validation set was used to control (and terminate) the training process, while final accuracy was estimated using the test set. All single images were placed in the training set, so that the testing and validation sets only contained paired images.

Augmentation is an important technique for training deep CNNs on limited data sets [9]. This process applies a set of random transformations that preserve class, whilst artificially inflating the training data set size. Therefore, the classifier is unlikely to encounter the exact same input twice, and is less likely to overfit the data i.e. learning to recognize individual input images, rather than identifying general features. We applied standard image augmentation to our data set using Keras and TensorFlow. The images were rotated randomly between 0 and 360 degrees, reflected by the vertical or horizontal axis, and vertically shifted by +/- 10 pixels. In addition, standard image normalization for CNNs was applied, mapping the 0-255 pixel values for each image to values between 0 and 1.

The configuration of the training process is determined by a set of hyperparameters. Batch size defines the number of images to be processed at a time during training, and the gradient of the error function for the current parameter is calculated for each batch. The optimizer function determines how the weights are modified from the gradient. Here, we used stochastic gradient descent (SGD), rmsprop, and Adam [27]. Weight decay is a regularization method that causes the weights to gravitate towards smaller values, limiting the nonlinear behavior of the classifier.

GridSearchCv from ScikitLearn [28] and KerasRegressor from Keras were used to perform a grid search of the hyperparameter values shown in Table 1. The optimal values of the hyperparameters were found using the Adam optimizer function [27], a batch size of 20, learning rate of 0.0004, and a decay value of 0. In addition, the patience, which controls termination of training, was set to 20, the epoch was set to 150, and steps was set to 1600. In total, a complete training run can process 4.8 million images.

**Table 1:** Hyperparameter configurations explored.

Hyperparameter	Values explored
Batch size	8, 12, 16, 20
Learning rate	0.1, 0.01, 0.0004, 0.0001
Optimizer	SGD, rmsprop, Adam
Weight decay	0.01, 0.001, 0

### Comparing accuracy to human experts

To compare the performance of the CNN model with that of human experts, we used the same method that is used when evaluating human versus human precision [29]. Since the actual age of the fish is unknown, the accuracy cannot be assessed and so the coefficient of variation (CV) of the (assumed) independent estimators is used. For a given otolith *j*, estimator *i* provides an age estimate *X*_*ij*_ for otolith *j*, and the CV for that individual otolith *j* is given as

$$\begin{array}{c} CV_{j}=\frac{\sqrt[{}]{\sum_{i=1}^{R}\frac{{\left(X_{ij}-X_{j}\right)}^{2}}{R-1}}}{X_{j}}, \end{array}$$ (2)

where *R* is the number of individual estimators and $X_{j}=\frac{1}{R}\sum_{i=1}^{R}X_{ij}$. To assess the overall performance across the otoliths for the full data set, the mean CV is used and defined as

$$\begin{array}{c} \bar{CV}=\frac{1}{J}\sum_{j=1}^{J}CV_{j}, \end{array}$$ (3)

where *J* is the number of otoliths.

To evaluate the CNN model, we estimated the CV using the CNN as one estimator (*i* = 1), and the human-read age as the other (*i* = 2), resulting in two individual estimators and hence, an R value of 2.

Since the CNN is reading both images, we used two different definitions of the CNN to read otoliths, i.e. two different definitions of the age estimate, *X*_1*j*_ (c.f. Eq (2)). The first definition, was derived from an average taken over an image pair, and is given by

$$\begin{array}{c} X_{1j}=\frac{X_{1j}^{\left[R\right]}+X_{1j}^{\left[L\right]}}{2} \end{array}$$ (4)

whereas the second definition only considers the right otolith, i.e. $X_{1j}=X_{1j}^{\left[R\right]}$, where $X_{1j}^{\left[L\right]}$ and $X_{1j}^{\left[R\right]}$ are the CNN-predicted age of the left and right otolith respectively. The first definition is based on our approach and the latter is based on what an expert reader would do [13] for this specific data set, and both were tested.

To evaluate the merit of CNN versus pure reader-based CVs, the latter was taken from the literature [13, 16]. A drawback of this approach is that any between-reader bias may affect the reader-based CV by an unknown amount.

## Results

Predictions were made on the test set for the different configurations and the MSEs of single otolith predictions of age were recorded. The MSE and CV of pair-wise predictions were also recorded. Calculated CV values were then used to select the optimum CNN model.

The MSE values of the predictions made for the left and right otoliths and both otoliths combined were 3.27, 2.71 and 2.99 respectively (Table 2). Using the average of the predictions for each of the paired otoliths resulted in the lowest MSE value (2.65).

**Table 2:** MSE (Eq 1) and mean CV (Eq 3) for predictions. The statistics are calculated on on single, left, right and paired (both left and right) otolith images.

Metric	Single	Left	Right	Paired
MSE	2.99	3.27	2.71	2.65
mean CV	9.47	9.97	8.97	8.89

**Fig 4:**
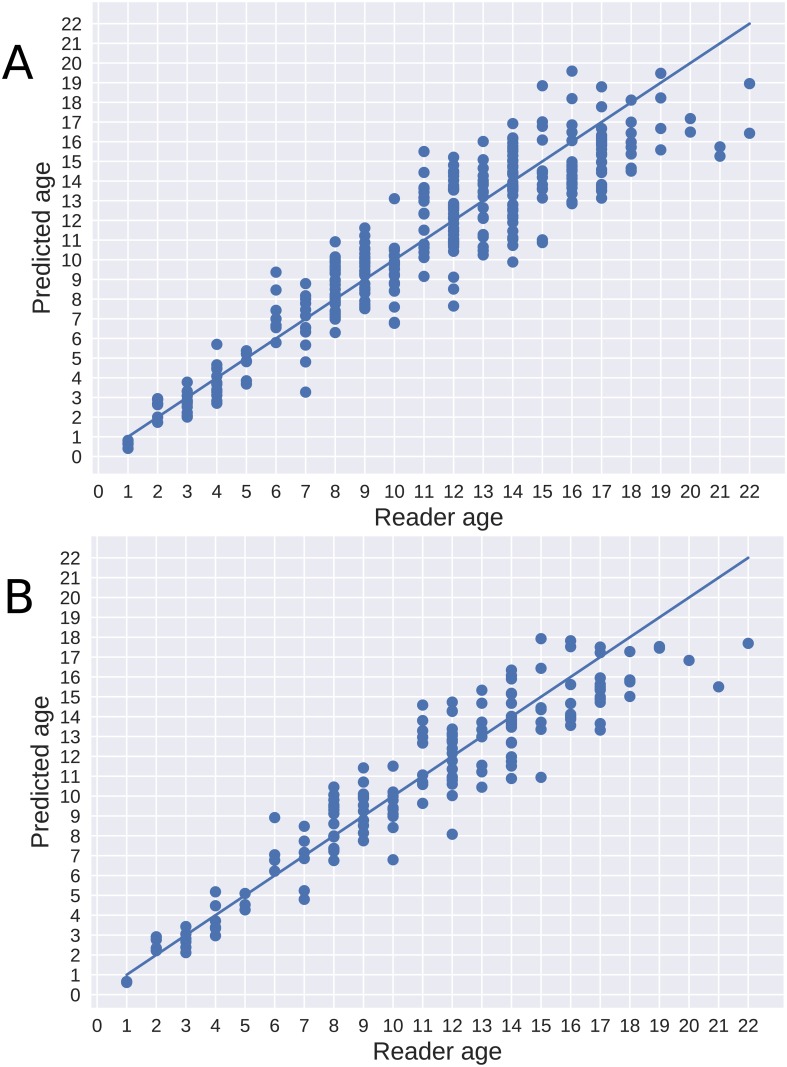
Age predictions. Predictions are shown using single otoliths (A) and using the average prediction of each pair (B), compared to the age estimated by a human reader.

Fig 4 shows that using both otoliths in an ensemble reduces prediction variance. There was also a clear tendency for the system to predict a lower age for older individuals, when compared to human readers. The variance of the predictions also increased with the age of the otolith.

**Fig 5:**
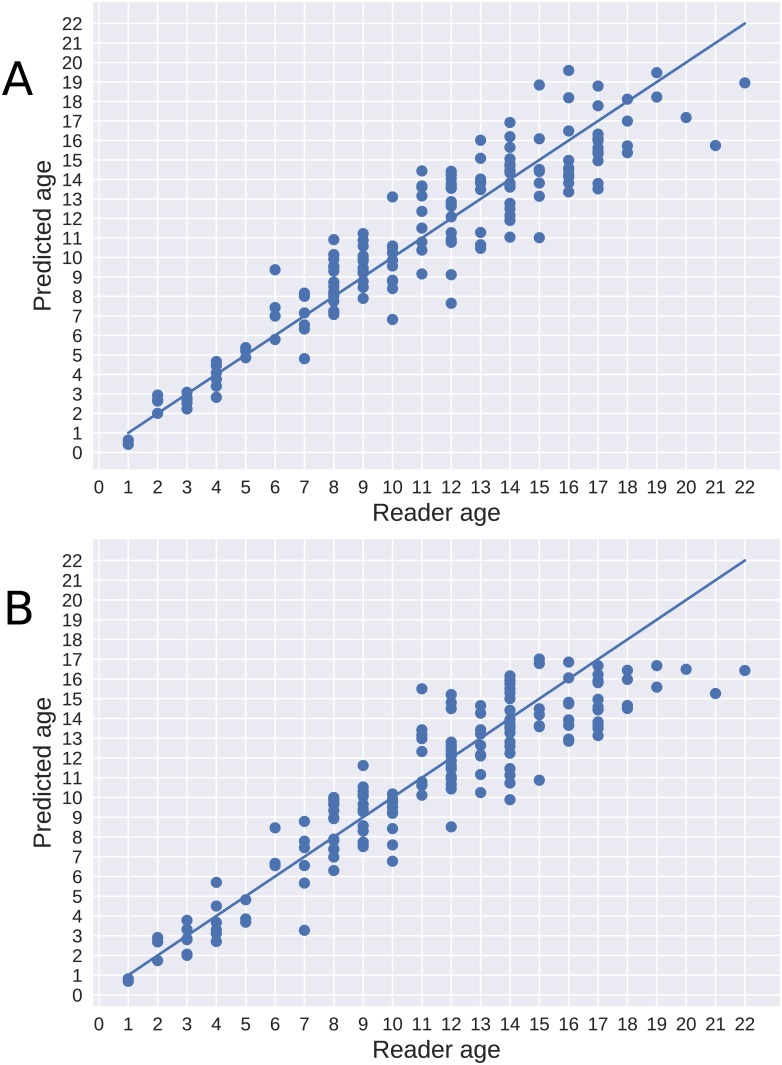
Age predictions. Predictions are shown for the right (A) and left (B) otoliths compared to the age as estimated by a human reader.

For Greenland halibut, the mean CV between human experts has previously been reported as 12% and 16.3% [13, 16]. Using otolith pairs, we achieved a mean CV of 8.89%. Fig 5 shows predictions for left and right otoliths separately. Age was correctly estimated for 48 out of the 164 tested otolith-pairs (29%). In addition, 63 cases (38%) were estimated to be one year off the read age.

## Discussion

The objective of this study was to investigate to what extent a deep CNN could be adapted to predict age from otolith images. Using a data set of Greenland halibut otoliths, we trained and validated an Inception-3 network and showed that it performed at a level close to human accuracy. Deep neural networks have been shown to outperform more conventional methods across a range of problems [9], and given their generality, we hypothesized that they would perform well on this rather difficult task. Several different network architectures were used, and most configurations were able to perform well, which further supports our hypothesis.

A simplistic approach was taken when preprocessing the images. Potentially informative properties, such as size, proportion and orientation, were lost through rescaling and augmentation, but this did not notably affect the network’s ability to predict age. The classifier functioned robustly across varying backgrounds. Traditionally, preprocessing algorithms have also been used to enhance features for the classifier. We also experimented with various preprocessing techniques. For example, we ran the images through a hill shading algorithm before training, but it did not improve results. This supports the conventional wisdom that deep neural networks are able to identify informative features directly, and that developing specialized preprocessing techniques is likely to be unnecessary.

We found a much stronger correlation between the otolith pixel area and CNN predicted age in the test set, than the correlation between pixel area and the human-read age. This indicates that size is a major feature associated with age in the CNN, despite the fact that the images of single otoliths, produced by splitting paired otolith images, varied in size and were rescaled by different proportions. Therefore, a future task of this work is to apply randomized scaling as an augmentation feature, to determine how sensitive the results are with regard to otolith size.

**Fig 6:**
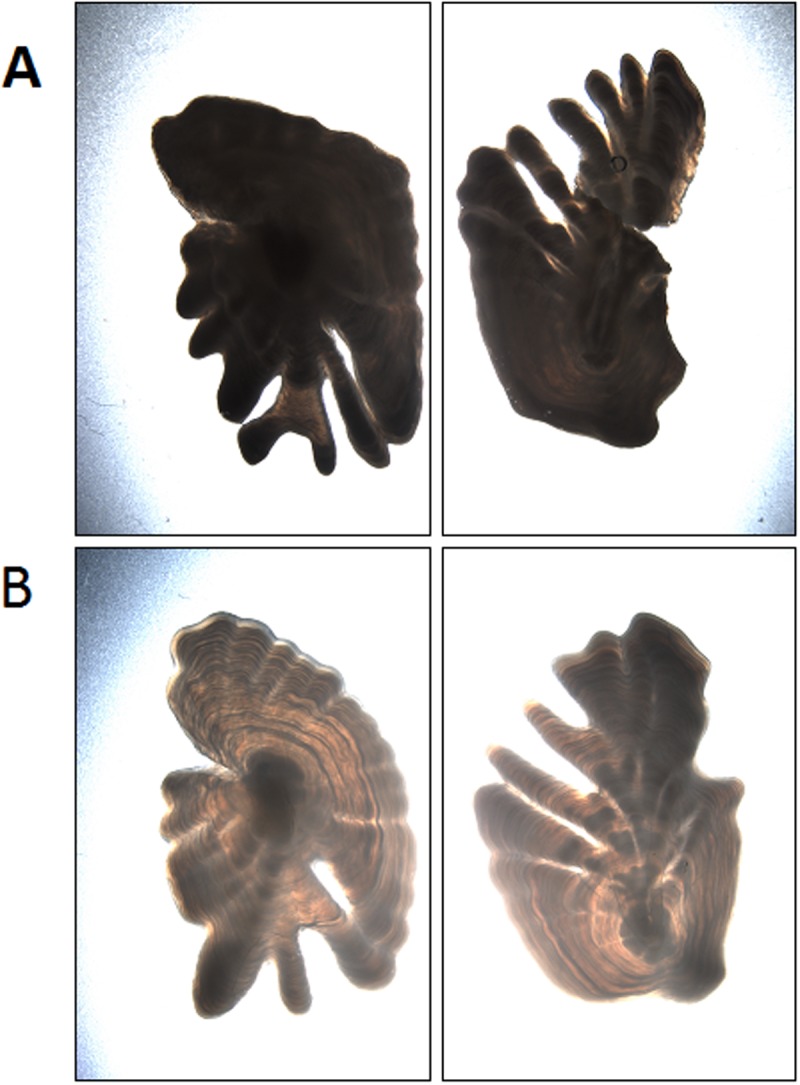
Examples of images where the network failed to correctly predict age. A) Dark images of otoliths with deep lobes are read as 12 years, and predicted as 15.7 (left), and 15.6 (right). B) Lighter otoliths below are read as 21 years, and predicted as 15.6 (left and right).

While we have not performed an extensive analysis of cases where the network failed to correctly predict age, a cursory inspection revealed that image inconsistencies (some examples are shown in Fig 6) could impact the results. This suggests that if the process of taking the images could be standardized, e.g. using consistent equipment, range, lighting conditions etc., then results could be improved.

The cost function applied was not adjusted for an imbalanced data set i.e. a prediction bias for the more abundant year classes would of been penalized more than classes with relatively lower abundances. This could explain the lower prediction accuracy for older otoliths, as there were relatively fewer otoliths from older fish. One way to mitigate this is by implementing a cost function that weights classes evenly i.e. each year class inflicts the same cost [30]. However, for ages that are critical to assessment, incorrect predictions should be associated with higher penalties.

Since the model is a supervised machine learning algorithm, the learning can only be as good as the underlying precision and accuracy. Since the accuracy is unknown [13], we treated the CNN as an individual reader and computed the same mean CV as is used in human versus human comparisons. We achieved a mean CV of 8.89%, which is considerably lower than the reported mean CV of human readings, ranging from 12 to 16.3% [13, 16]. However, a between-reader bias could have increased the reported CVs. In our case, each otolith in the test set was only read by one of two readers from the same lab. Therefore, it is reasonable to assume that in this case the bias is likely to be negligible. The large variation in reported CVs also indicates that this is a sensitive measure, and that not too much importance should be attached to our relatively low CV.

A common criticism of CNNs is that the exact features used in the process are unknown. During the training and testing of the CNN, we set aside 4% of the data set for validation (during training), and 4% for testing (after training), meaning that 8% of the data was not used to train the network. However, when the method is in production, it is important to keep validating the method by continuing to collect training data. This is particularly important if the method is used as part of a monitoring time-series.

Using CNNs to make age predictions can be more efficient than expert-read predictions. If cost savings are the key motivation for implementing automated aging of otoliths, a common objection is that any cost savings relies on the assumption that the actual reading is the factor that drives the cost. In reality, the time required for otolith preparation, i.e. removing the otoliths and preparing the sample for imaging, may take more time than the actual reading, and so the savings would be marginal. However, skilled readers require years of training, which should be considered when determining cost. Assuming that the current staff is maintained and used to generate validation data, the sampling program could be scaled up without the necessity to train more readers. Furthermore, if the network is indeed able to learn characteristics of individual readers, it is possible to explore downstream effects of using different readers, as well as interpreting otoliths using an ensemble that emulates multiple readers for increased accuracy.

In their recent rewiew, Fisher and Hunter [31] found that digital image analysis systems provided little improvement in cost-effectiveness over manual otolith analysis. Although machine learning systems were included in their study, no modern deep learning convolutional network was considered. In light of the accuracy we have demonstrated from such a system, this conclusion may need to be revised.

Future work should include testing the method on other species and new features. The method could also be adapted to specific use cases or enhanced by other predictors. Organizing data and collecting images and age labels for a wider range of species is required to move forward. It is likely that the patterns used to age Greenland halibut are similar to the general patterns for other species, which makes the classifier ideal for using transfer learning. Furthermore, a general CNN could be trained using otolith images from multiple species, and then fine-tuned to each specific species. Other features like age-at-maturation (spawning zones) could also be read from otoliths, and where training data are available, the network could be adjusted to predict these features as well.

### Conclusion

Age determination from otoliths is an important input for management of marine fish stocks. Here, we predicted age of Greenland halibut by training a CNN using otolith images. The results indicate that automating the data processing for this intrinsically complicated process is possible. On top of its ability to learn aging, the method offers improved efficiency, the possibility to learn how to read otoliths across species, and, given proper attention to the collection of validation data, increased consistency over time. Since age is an essential component of any age-based model, the method will have an impact on the management of fish resources and our understanding of ecosystem dynamics.
